# The secreted host-cell protein clusterin interacts with PmpD and promotes *Chlamydia trachomatis* infection

**DOI:** 10.3389/fcimb.2024.1519883

**Published:** 2025-01-27

**Authors:** Fabienne Kocher, Johannes H. Hegemann

**Affiliations:** Institute for Functional Microbial Genomics, Faculty of Mathematics and Natural Sciences, Heinrich Heine University Düsseldorf, Düsseldorf, Germany

**Keywords:** PmpD adhesin, secreted human clusterin, interaction, *Chlamydia trachomatis*, infection

## Abstract

Attachment and uptake into host cells are pivotal steps in the life cycle of the *Chlamydiaceae*, a family of obligate intracellular pathogens. *Chlamydia trachomatis (Ctr)* possesses a family of nine polymorphic membrane proteins (Pmps), which have been shown to be crucial for adhesion and internalization. However, the host-cell molecules involved have so far remained unknown. Here, we show that a fragment of *Ctr* PmpD, which forms high-molecular-weight oligomers in solution and adheres to epithelial cells, also binds to secreted clusterin (sCLU), a chaperone-like protein that is secreted into the extracellular space by the host cell, and forms part of the chaperone- and receptor-mediated extracellular protein degradation (CRED) pathway. Using *in vitro* assays, we demonstrate that sCLU interacts directly with soluble rPmpD. In infection experiments, depletion of sCLU from the culture medium leads to a significant decrease in *Ctr* infection. Thus, sCLU is the first host-cell interaction partner identified for a *Ctr* Pmp and the first case in which sCLU has been shown to be a vital component for the establishment of a bacterial infection.

## Introduction

*Chlamydia trachomatis* (*Ctr*) is the most common bacterial cause of sexually transmitted infections worldwide. If untreated, it can lead to infertility or ectopic pregnancies (Rodrigues et al., 2022). In addition, *Ctr* is the leading cause of trachoma, the world’s primary cause of infectious blindness, which has been targeted for elimination by the end of the decade (Malecela and Ducker, 2021). *Ctr* belongs to the genus *Chlamydia*, all of which are obligate intracellular, Gram-negative bacterial pathogens that infect epithelial cells (Elwell et al., 2016). Most chlamydial species are pathogenic to animals. However, two species, *Ctr* and *Chlamydia pneumoniae* (*Cpn*) – which infects the respiratory tract – are pathogenic to humans (Kalman et al., 1999). *Ctr* can be subdivided into 19 serovars and are grouped into three biovars, each of which is responsible for specific pathological conditions (Murray and McKay, 2021). Serovars A-C lead to trachoma and blindness (Murray and McKay, 2021). Serovars D-K cause infections of the genital tract and can lead to pelvic inflammatory disease (PID), ectopic pregnancy and infertility in women, and urethritis and epididymitis in men (Murray and McKay, 2021). Serovars A-K typically cause local infections only. Serovars L1-L3, also known as the lymphogranuloma venereum biovar, lead to invasive infections of the urogenital and anorectal tract, and cause systemic infections (de Vrieze and de Vries, 2014).

All chlamydial species, including *Ctr*, utilize a unique type of developmental cycle that involves switching between two morphological forms – the infectious elementary body (EB) and the non-infectious reticulate body (RB) (Moulder, 1991). Chlamydial infections are initiated when EBs adhere to, and are taken up by host cells. After internalization, the EB remains in a membrane-enveloped compartment known as an inclusion, in which it differentiates into an RB. By hijacking nutrients from the host cell, the RB undergoes repeated replication cycles before the pool of RBs asynchronously transitions back into EBs, which are released into the extracellular space either via cell lysis or extrusion of the inclusion (Moulder, 1991; Abdelrahman and Belland, 2005; Elwell et al., 2016; Christensen et al., 2019).

For obligate intracellular pathogens, adhesion to and uptake into the host cell is of the utmost importance. For the successful implementation of these processes, *Chlamydiae* have evolved a range of adhesins including GroEL1, MOMP, OmcB, Ctad1 and a whole family of polymorphic membrane proteins (Pmps) (Stephens et al., 2001; Mölleken and Hegemann, 2008; Wuppermann et al., 2008; Hegemann and Moelleken, 2012; Fechtner et al., 2013; Becker and Hegemann, 2014; Stallmann and Hegemann, 2016).

Pmps are synthesized by all chlamydial species and can be subdivided into six phylogenetic subtypes (A to H), with 21 members in *Cpn* and 9 members in *Ctr* (Grimwood and Stephens, 1999). Pmps share between 19% and 40% sequence identity across species, and are thought to belong to the broader class of type V autotransporters, based on their domain architecture (Henderson and Lam, 2001). These proteins possess an N-terminal secretion signal, a C-terminal β-barrel and a central passenger domain (PD) (Henderson and Lam, 2001). The signal sequence triggers the Sec-dependent transportation of the Pmp(s) across the inner membrane into the periplasmic space, where the Sec sequence is typically split off and the protein folds. The β-barrel is inserted into the outer membrane, supported by the BAM complex, and forms a channel that enables the PD to be exported to the cell surface (Henderson et al., 2004). The available evidence suggests that the extracellular Pmp PDs exist in both membrane-anchored and soluble forms, for which proteolytic processing sites have been identified (Wehrl et al., 2004; Kiselev et al., 2007; Swanson et al., 2009). Furthermore, Pmp PDs are exceptionally rich in FxxN and GGA(I,L,V) motifs, which have been shown to be crucial for the adhesion of EBs to epithelial cells (Mölleken et al., 2010).

Among the nine *Ctr* Pmps, all of which are known to adhere to epithelial cells and are essential for infection (Becker and Hegemann, 2014), PmpD and its *Cpn* homologue Pmp21 are the best studied at present. Proteomic studies suggest that PmpD undergoes proteolytic processing, which results in several fragments with or without the β-barrel domain (Kiselev et al., 2009; Swanson et al., 2009). Interestingly, immunoaffinity-purified PmpD from the EB surface is organised into high-molecular-weight complexes, consisting of four to six PmpD fragments (Swanson et al., 2009). *In vitro*, the formation of such high-molecular-weight oligomers has been demonstrated for *Ctr* PmpA, PmpD, PmpG and PmpI, and they appear to be important for adhesion to epithelial cells (Favaroni and Hegemann, 2021). Biochemical studies have revealed that the monomeric PmpD PD has a high β-sheet content and probably folds into a triangular β-helical structure (Paes et al., 2018; Cervantes et al., 2024). However, oligomeric forms of the PmpD PD have been suggested to form fibril-like structures, like those formed by the amyloid protein fragment Aβ42 that is associated with Alzheimer’s disease (Gu and Guo, 2013; Paes et al., 2018; Favaroni and Hegemann, 2021). Functional studies have shown that PmpD makes a critical contribution to the infection process. Anti-PmpD antibodies exhibit significant pan-neutralizing activity against a number of different *Ctr* serovars in cell culture, and a PmpD-based vaccine has shown protective activity in mice (Crane et al., 2006; Paes et al., 2016). In addition, a *pmpD* null mutant of *Ctr* serovar D showed significantly reduced adhesion and internalization capacities in human cell lines and non-human primate models, but not in murine cells (Kari et al., 2014). Interestingly, in *C. muridarum*, transposon-mediated inactivation of PmpD, PmpA or PmpI leads to growth attenuation and reduced the numbers of infectious progeny, suggesting that these proteins may have other functions in addition to adhesion (Wang et al., 2019). For the *Ctr* PmpD homologue in *Cpn*, Pmp21, similar structural and functional characteristics have been reported (Mölleken et al., 2010; Mölleken et al., 2013; Luczak et al., 2016). However, the roles of PmpD and Pmp21 in infection are thought to be species-specific, as recombinant Pmp21 reduces *Cpn* but not *Ctr* infection, and *vice versa* (Becker and Hegemann, 2014). Pmp21 binds to and activates the epidermal growth factor receptor (EGFR), which facilitates chlamydial uptake via endocytosis. Thus, Pmp21 functions as both an adhesin and an invasin (Mölleken et al., 2013). Similarly, *C. psittaci* Pmp17G, which belongs to a different subtype than either *Ctr* PmpD or *Cpn* Pmp21, is also suggested to bind the EGFR (Li et al., 2021). Importantly, while several human cell-surface receptors, such as heparan sulfate proteoglycans, CFTR, β1-integrin, ephrin A2, and protein disulphide isomerase have been associated with adhesion and/or internalization of *Ctr*, no receptor has been identified for *Ctr* Pmps to date (Romero et al., 2020).

Here, we have used a proteolytically processed form of PmpD from *Ctr* serovar E, found in both soluble and membrane-bound complexes during infection, to identify its host-cell binding partner(s) (Swanson et al., 2009). *Ctr* Serovar E is the most commonly identified subtype in clinical cases (Lesiak-Markowicz et al., 2019). Biochemical characterization of this PmpD fragment, called rD72, revealed that it forms high-molecular-weight oligomers, and binds to epithelial cells in a concentration-dependent manner. Incubation of rD72 with human epithelial HEp-2 cells, followed by immunoprecipitation, affinity enrichment and mass spectrometric analysis revealed that rD72 interacts with the secreted form of the host-cell protein clusterin (CLU). Clusterin is a human protein, part of which is secreted into the extracellular space, where it facilitates the *in-vivo* clearance of misfolded extracellular proteins, with a high preference for amyloid-β-like structures. Secreted CLU (sCLU) is known for its chaperone-like function; it binds to its cargo protein, and induces uptake of the complex into the target cell, where it is eventually degraded. sCLU also acts as a terminal pathway inhibitor of the complement system by interacting with that system’s membrane-attacking complex (MAC) (Choi et al., 1989). Using biochemical and biological approaches, we have confirmed the direct interaction of PmpD with sCLU. Furthermore, we demonstrate that the absence of sCLU in the culture medium significantly reduces *Ctr* infection in epithelial cells. In summary, we show that *Ctr* PmpD binds to secreted clusterin, and that the presence of extracellular sCLU is essential for a *Ctr* infection.

## Results

### D72, a proteolytically processed fragment of PmpD, forms high-molecular-weight homo-oligomers and binds to epithelial cells

**Figure 1:**
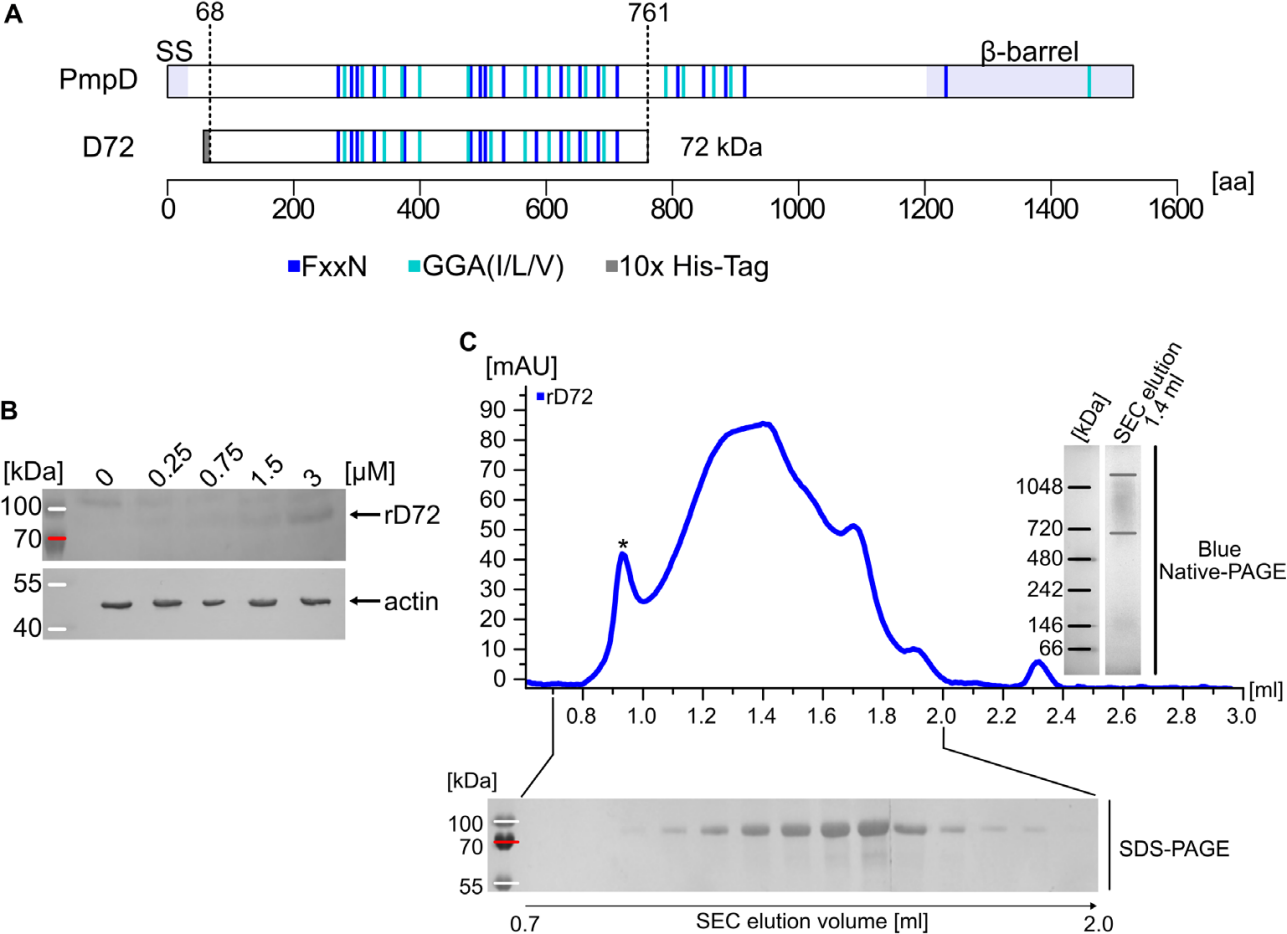
Recombinant D72 is a biologically functional fragment of *Ctr* PmpD. **(A)** Schematic representation of *Ctr* PmpD and its proteolytically processed fragment D72, which spans aa residues 68-761. The N-terminal signal sequence (SS), the passenger domain (PD) and the C-terminal β-barrel are indicated. The positions of FxxN and GGA(I,L,V) motifs are marked in dark and light blue, respectively. The grey box indicates the genetically fused 10x His-Tag. The processed fragment includes 14 FxxN and 12 GGA(I,L,V) motifs. The image was generated in R Studio using a customized code (RStudio Team, 2020; R Core Team, 2024). **(B)** Increasing concentrations of His-tagged soluble recombinant D72 (rD72) was added to the cell culture medium (ccm) and incubated for 1 h at 4°C with HEp-2 cells, which were grown in this ccm for two days. Bound protein was visualized on a Western blot using an anti-His antibody. Actin was used as an internal loading control. **(C)** Size-exclusion chromatography (SEC) was used to analyse the oligomeric state of rD72 in solution. The void volume is indicated by an asterisk. Samples of the individual elution fractions were analysed by SDS-PAGE (bottom). A sample of the elution fraction at 1.4 ml was also collected and analysed by Blue Native-PAGE (right).

Previous work showed that all nine *Ctr* Pmps can bind to epithelial cells and block subsequent *Ctr* infection; this is why Pmps were defined as adhesins (Becker and Hegemann, 2014). The Pmp fragments used in those studies were deliberately designed to focus on regions with high densities of GGA(I,L,V) and FxxN motifs, since these characteristic tetrapeptide motifs had initially been shown to be essential for adhesion (Mölleken et al., 2010). Here, we focus on D72, a fragment of the *Ctr* PmpD, which was discovered in *in-vivo* studies (**Figure 1A**) (Kiselev et al., 2009; Swanson et al., 2009). D72 spans the N-terminal half of the passenger domain (PD) of PmpD, which encompasses amino-acid residues 68 to 761. It therefore includes 12 GGA(I,L,V) and 14 FxxN motifs. Structure prediction tools suggest that the recombinant D72 (rD72) forms a lengthy β-helical structure, as has been suggested for fragments of other Pmp PDs (**Supplementary Figure S1**) (Debrine et al., 2023).

In order to characterize the biochemical and functional properties of rD72, its ability to (i) adhere to epithelial cells and (ii) form high-molecular-weight oligomers was investigated. To this end, D72 was expressed in *E. coli* and purified with the aid of an N-terminal 10x His-Tag (rD72, **Figure 1A**). Adhesion to epithelial cells was tested by adding different concentrations of soluble rD72 to human HEp-2 cells growing in cell culture medium (ccm). Binding of rD72 to HEp-2 cells was detectable even at the lowest concentration used (0.25 µM). With increasing concentration of rD72 in the ccm, binding of rD72 also increased (**Figure 1B**).

Pmp protein fragments are known to form homo- and hetero-oligomers *in vitro* (Swanson et al., 2009; Luczak et al., 2016; Paes et al., 2018; Favaroni and Hegemann, 2021). The oligomerisation of rD72 was investigated by size-exclusion chromatography (SEC), Blue Native-PAGE (BN) and SDS-PAGE (**Figure 1C**). Soluble rD72 was loaded onto a Superose 6 SEC column, and was eluted in a broad peak, which began to rise after the column void volume and eventually spanned one-third of the total volume of the column bed (**Figure 1C**). SDS-PAGE verified the presence of full-length rD72 (**Figure 1C**, *bottom*). BN analysis of the SEC elution fraction at 1.4 ml revealed a high-molecular-weight oligomer of between 720 and 1048 kDa, corresponding to a calculated 10- to 15-mer (**Figure 1C**, *right*).

Taken together, these data indicate that rD72 is a biologically functional PmpD fragment that adheres to epithelial cells. In addition, rD72 shows comparable biochemical characteristics to those of other Pmp PD fragments, including a high degree of oligomerisation in solution (Luczak et al., 2016; Paes et al., 2018; Favaroni and Hegemann, 2021). Based on these findings, rD72 was used in further experiments with the aim of identifying host-cell binding partner(s).

### Secreted clusterin is a host-cell binding partner for rD72

**Figure 2:**
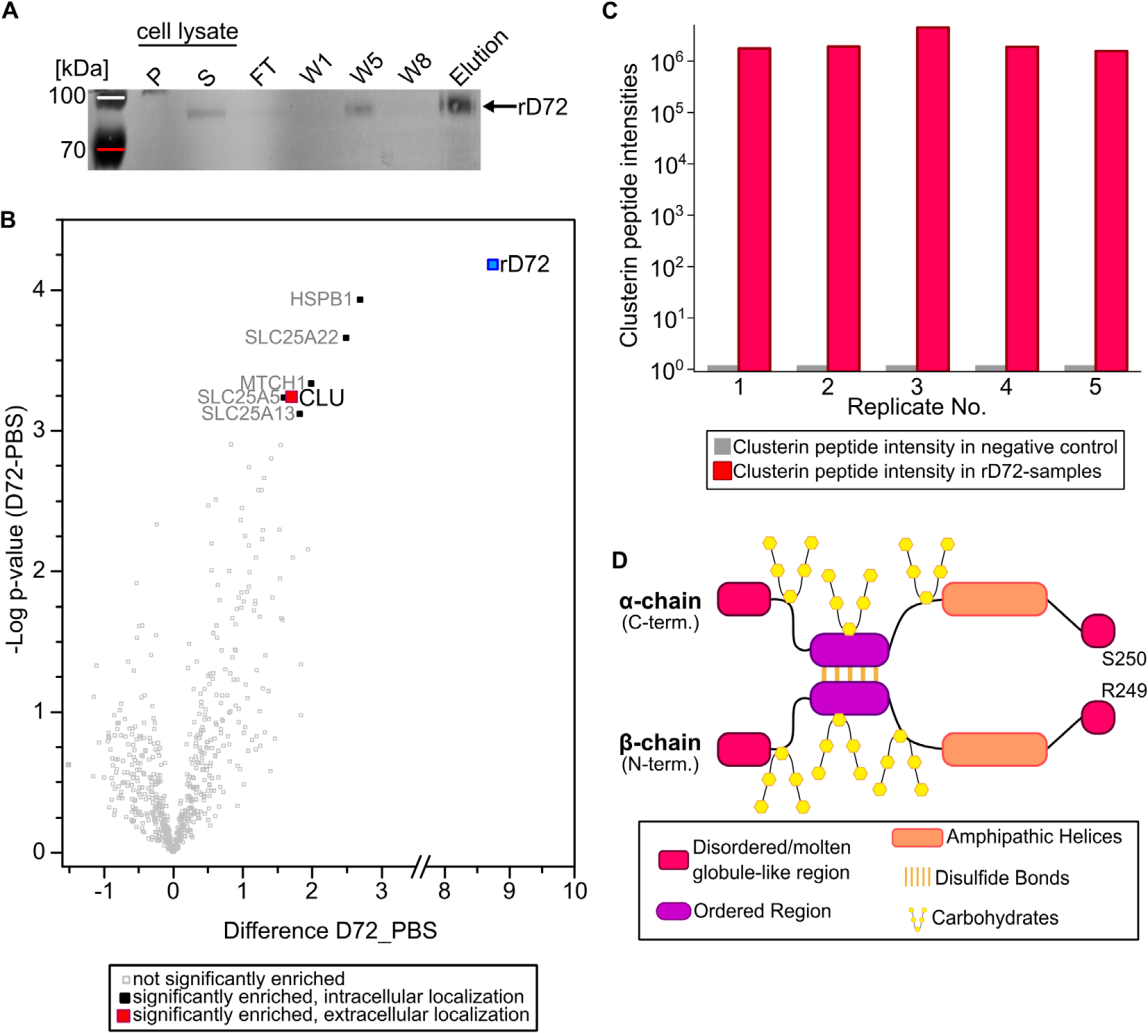
rD72 binds to the secreted host-cell chaperone clusterin. **(A)** Pulldown experiment in which rD72 was added to HEp-2 cells which had been grown for 2 days in cell culture medium (ccm). Cells were lysed to solubilize protein complexes and the lysate was cleared by centrifugation (P = pellet, S = supernatant). rD72 and any bound host-cell interaction partner(s) were co-purified using Ni-NTA agarose. Fractions of the individual Ni-NTA purification steps were analysed on Western blots, and probed with an anti-His antibody. (FT = flow-through, W1 = wash 1, W5 = wash 5, W8 = wash 8) **(B)** Volcano plot of human proteins identified by mass spectrometry after affinity purification (n=5). Proteins that were significantly enriched relative to the negative control (PBS) are labelled (Uniprot nomenclature). The fold change (rD72 minus PBS) is plotted against the difference in mean values of log_2_ label-free quantitation (LFQ) intensities (rD72 minus PBS). **(C)** The intensities of clusterin peptides in each replicate are compared between the test sample containing rD72 (red columns) and the corresponding negative control (PBS, grey columns). **(D)** Schematic representation of secreted human clusterin (sCLU). In the cytoplasm, the clusterin precursor is N-glycosylated and is cleaved between residues R249 and S250. The resulting α- and β-chains are then linked in an antiparallel fashion by five intramolecular disulfide bonds, thus generating the secreted isoform (sCLU). Mature sCLU has a molecular weight of ~70 kDa and is secreted into the extracellular space.

We carried out immunoprecipitation experiments to identify potential host-cell binding partner(s) for rD72. To this end, confluent HEp-2 cells grown in cell culture medium (ccm) were supplemented with soluble rD72 and incubated for 1 h at 4°C. As the negative control, fresh PBS was added to the ccm. After 1 h, cells were washed three times with HBSS to remove unbound rD72, then cells were lysed, and rD72-containing complexes were isolated by affinity purification (AP) using Ni-NTA agarose. The individual purification steps were monitored via Western-blot analysis, using anti-His antibodies to detect rD72 (**Figure 2A**; **Supplementary Figure 3**). The protein composition of the elution fraction, containing rD72, was then subjected to mass spectrometric analysis to identify proteins that co-eluted with rD72.

Intriguingly, apart from rD72 itself, only five significantly enriched host cell proteins were detected in the AP elution fraction (**Figure 2B**; **Supplementary Figure S2**; **Supplementary Table S2**). All five of these were identified as intracellular proteins, and thus should not be accessible for interactions with extracellular rD72. Indeed, four of them are predicted to be mitochondrial components (**Supplementary Table S2**). However, the fifth, clusterin (CLU), is also known to be secreted in a soluble extracellular form, designated as sCLU, and is therefore a potential candidate binding partner for rD72 (Bettens et al., 2015). Detailed analysis of the CLU peptide intensities detected by mass-spectrometric analysis confirmed that CLU was enriched in all five replicates of the test samples, and was not found in the negative controls (**Figure 2C**).

CLU is an ATP-independent chaperone whose main function is to stabilise misfolded proteins and inhibit the formation of amorphous or amyloid-like aggregates, particularly in the extracellular space (Poon et al., 2000; Hatters et al., 2001). The sCLU isoform is derived from a precursor polypeptide of ~50 kDa, which undergoes extensive post-translational modification, including extensive glycosylation (Gross et al., 2023). Furthermore, sCLU forms five intramolecular disulphide bonds, which connect the α- and β-chains in an antiparallel arrangement following cleavage of the precursor molecule (**Figure 2D**) (Burkey et al., 1991). The fully glycosylated α- and β-chains have molecular weights of 34-36 kDa and 36-39 kDa, respectively (de Silva et al., 1990).

### *In vitro* pulldown assays verify the interaction between rD72 and sCLU

**Figure 3:**
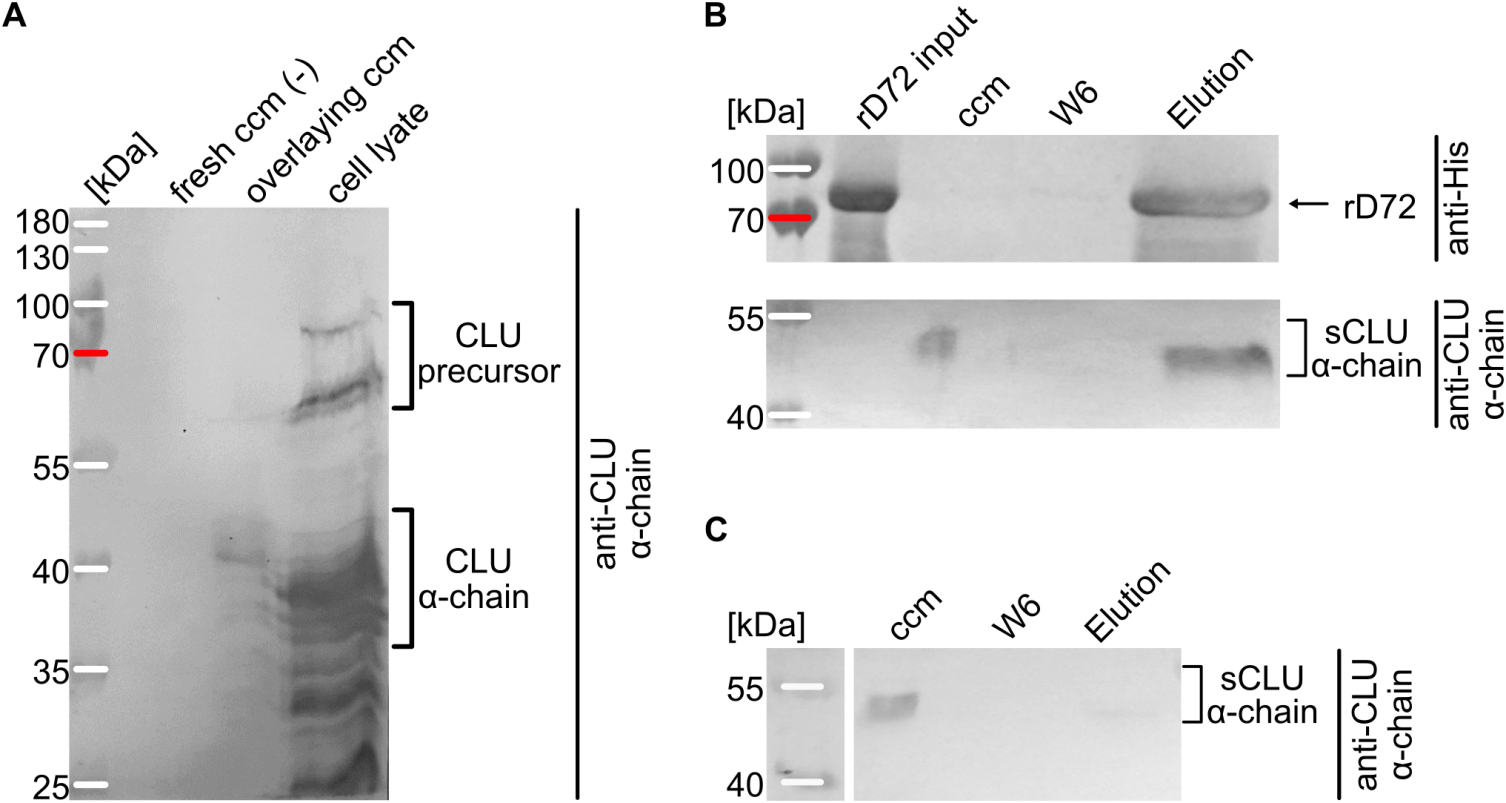
*In vitro* interaction of rD72 with clusterin. **(A)** Analysis of clusterin abundance in HEp-2 cells by Western-blot analysis, using an antibody against the clusterin α-chain. Fresh cell culture medium (ccm) was used as the negative control (-). In the ccm isolated from cells that had been grown for 2 days (overlaying ccm), the post-translationally modified clusterin α-chain is detected at ~40-45 kDa (theoretical MW: 36-39 kDa). In the cell lysate, bands appear at 60-80 kDa (corresponding to the clusterin precursor) and the bands at ~40-45 kDa represent the fully processed α-chain of secreted clusterin (sCLU). **(B)** Ni-NTA pulldown assays. rD72 was incubated with ccm isolated from epithelial cells that had been grown for two days, and used as bait. The protein composition of the individual fractions of the pulldown assay was probed after Western blotting, using anti-His and anti-clusterin α-chain antibodies. (W6 = wash 6). **(C)** Negative control for the Ni-NTA pulldown assay. The ccm used was obtained from HEp-2 cells that had been grown for two days in the absence of rD72. Fractions were separated by SDS/PAGE and analysed via Western blot, using an anti-clusterin α-chain antibody.

To investigate the respective levels of intra- and extracellular CLU, HEp-2 cells were seeded in sub-confluent amounts and grown for two days at 37°C in ccm. The ccm and the cultured cells were then separately analysed by Western blotting with an antibody raised against the clusterin α-chain (**Figure 3A**). Fresh ccm served as the negative control, and no non-specific signals were observed. In contrast to this, ccm obtained from HEp-2 cells grown for two days was found to contain a broad protein band ranging in size from approximately 40 kDa to 45 kDa, which corresponded to the secreted CLU α-chain (**Figure 3A**). Multiple bands were observed in the cell lysate, representing the cleaved and uncleaved clusterin precursor, which had undergone different degrees of post-translational modification (**Figure 3A**). Thus, growing HEp-2 cells secrete mature clusterin into the extracellular medium.

To verify the direct interaction between sCLU and rD72, an *in vitro* pulldown assay was performed, using rD72 as bait (**Figure 3B**; **Supplementary Figure 4**). Supernatant obtained from HEp-2 cells that had been grown for two days was collected and incubated with soluble rD72. rD72 was then affinity-purified using Ni-NTA agarose, and the elution fractions were analysed on a Western blot. As a negative control, 2-day-old ccm was incubated with Ni-NTA agarose in the absence of rD72 (**Figure 3C**). While the negative control showed no non-specific binding of sCLU to the agarose matrix, the test samples consisting of ccm with the His-tagged rD72 as bait clearly demonstrated co-elution of both proteins in the elution fraction. These findings confirm that sCLU can interact with rD72.

### Depletion of secreted clusterin from the culture medium inhibits infection by *C. trachomatis*

**Figure 4:**
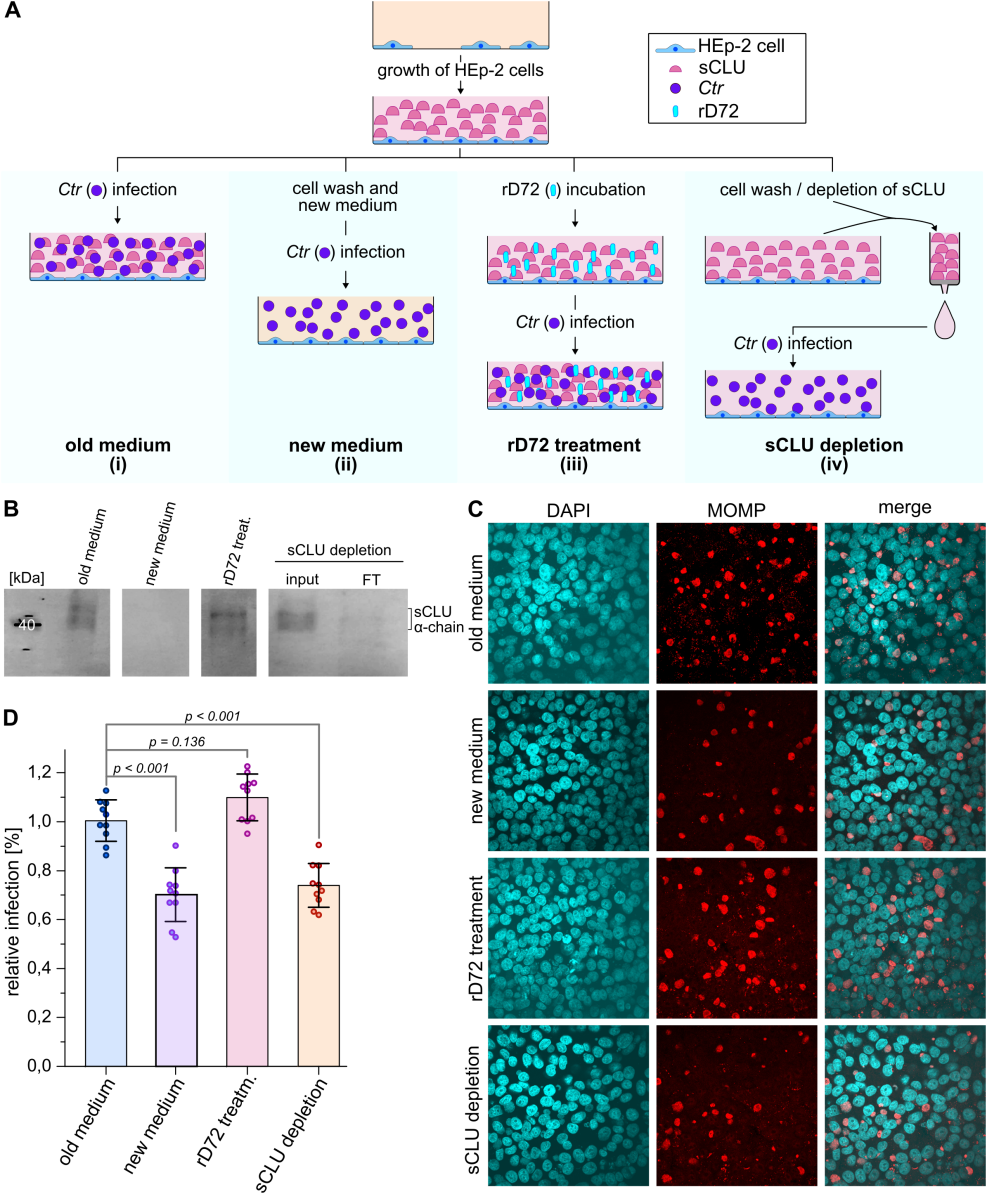
sCLU facilitates uptake of *Ctr* into host cells. **(A)** Schematic representation of the different experimental setups used to test the effect of sCLU during the initial steps of a chlamydial infection. In all four experiments (i-iv), confluent cells were grown for two days in ccm. In approach (i), *Ctr* EBs were added to confluently grown cells. For approach (ii), the cells were washed and fresh ccm supplemented with *Ctr* EBs was added, in the absence of sCLU. For approach (iii), the ccm was supplemented with soluble rD72 and incubated with HEp-2 cells for 1 h before *Ctr* EBs were added to the ccm. For approach (iv), ccm that had been used for cell growth was isolated and sCLU was extracted via affinity purification (AP), while the flow-through, depleted of sCLU, was returned to the HEp-2 cells, supplemented with *Ctr* EBs. For all four approaches, EBs were incubated with HEp-2 cells for 2 h at 37°C before the free EBs were removed. After 24 h, the infection was stopped by fixing and permeabilizing the cells with PFA and methanol. **(B)** Western blot analysis of ccm, using an anti-clusterin α-chain antibody. For approaches (i) to (iii), samples were taken from the ccm immediately prior to supplementation with *Ctr* EBs. For approach (iv), the AP input (input) and the AP flow-through (FT) were analysed. **(C)** Sections of immunofluorescence microscopy pictures of the individual infection approaches as depicted in **(A)**. DNA is visualized with DAPI (cyan) and inclusions are visualized with an anti-MOMP antibody (red). Images are representative of three biologically independent replicates (n=3). **(D)** Infection rates of the individual approaches (i-iv) were determined by calculating the ratio of inclusions to the number of HEp-2 cells. The infection rate of approach (i) was set to 1 and approaches (ii-iv) were normalized to it. Three biologically independent experiments (n=3) were performed, and for each replicate, 3-4 images on different sections were taken. The means of the individual data points are indicated by the height of the individual bars (± SD) and the *p*-values are indicated.

Having verified that sCLU interacts directly with *Ctr* PmpD, the role of sCLU during the initial stages of a chlamydial infection was investigated. To this end, HEp-2 cells were grown in ccm for two days, and the level of clusterin in the ccm was manipulated, prior to infection, using four different experimental approaches (**Figure 4A**). We first set up a regular infection experiment that served as a control (i). In this case, *Ctr* EBs were added to HEp-2 cells, which had been grown in ccm for 2 days, yielding “old medium” enriched in sCLU (**Figures 4A, B**). In the second approach (ii), ccm in which cells had been grown for two days was removed and replaced by fresh medium that was devoid of sCLU (“new medium”, **Figure 4A, B**), which was then supplemented with *Ctr* EBs to initiate infection. In the third approach (iii), after two days of cell growth, soluble rD72 was added to sCLU-rich “old medium” and incubated for 1 h before *Ctr* EBs were added (“rD72 treatment”, **Figure 4A**). Western-blot analysis showed no significant change in sCLU abundance after 1 h incubation with rD72, relative to the level of sCLU employed in approach (i) (**Figure 4B**; **Supplementary Figure 5**). In the fourth approach (iv), “old medium” in which cells had been grown for two days was isolated, and the sCLU contained in it was removed by affinity purification (AP). The flow-through fraction, depleted in sCLU, was added to the cell monolayer, which was then infected with *Ctr* EBs (**Figures 4A, B**).

In all cases, EBs were allowed to adhere to epithelial cells for 2 h prior to removal of the remaining free EBs, and the infection was allowed to proceed for 24 h (**Figure 4C**). Interestingly, the level of infection detected in cells grown in the presence of “fresh medium” that initially contained no detectable sCLU (approach (ii)) was found to be significantly reduced (by 30%) relative to the control experiment (i) (**Figure 4D**). Virtually the same reduction in the infection rate (to 0.72) was observed in approach (iv), in which “old medium” that had been depleted of sClu was used (**Figure 4D**). In contrast, pre-incubation of “old medium” (abundant in sClu) with rD72 in approach (iii) led to a modest increase in the infection rate (1.1) compared to the control (i) (**Figure 4D**). Importantly, we did not see any obvious differences in the inclusion size in the infection assays (i) to (iv).

The results obtained from approaches (i) to (iv) indicate the relevance of the availability of secreted clusterin for successful *Ctr* infection.

## Discussion

The initial adhesion to, and subsequent uptake of EBs into epithelial host cells are critical steps in *Ctr´s* developmental cycle. The primary contact between pathogen and host is mediated by chlamydial adhesins including GroEL1, MOMP, OmcB, Ctad1 and Pmps that bind to specific host-cell proteins (Mölleken and Hegemann, 2008; Wuppermann et al., 2008; Mölleken et al., 2010; Stallmann and Hegemann, 2016; Romero et al., 2020). The family of Pmps plays a complex but crucial role in the adhesion step and the ensuing internalization processes (Vasilevsky et al., 2016; Romero et al., 2020). All nine *Ctr* Pmps have been demonstrated to adhere to epithelial cells, and infection-blocking assays using soluble recombinant Pmp fragments have indicated that each of these proteins contributes to *Ctr* infectivity. However, their host-cell binding partners have remained unknown up to now (Becker and Hegemann, 2014). Here, we show that *Ctr* PmpD, which forms high-molecular-weight (hMW) oligomers in solution and attaches to epithelial cells, interacts with the secreted host-cell chaperone clusterin (sCLU). Under the experimental conditions used, infectivity is significantly reduced in the absence of sCLU in the cell culture medium (ccm) during the early stages of *Ctr* infection.

A fragment of the central passenger domain of PmpD, referred to here as rD72, can be found *in vivo* during infection (Swanson et al., 2009), and forms hMW oligomers in solution, as indicated by SEC analysis and Blue Native-PAGE (**Figure 1**). The SEC elution profile for rD72 resembles that of the 65-kDa PmpD PD fragment investigated by Paes et al (Paes et al., 2018). Similarly, Favaroni et al., using yet another motif-rich 66-kDa PmpD PD fragment, obtained similar results and further suggested, based on transmission electron microscopy, that these hMW oligomers might form protofibril-like structures (Favaroni and Hegemann, 2021). Furthermore, Luczak et al. working on Pmp21, the *Cpn* homologue of *Ctr* PmpD, showed that the hMW oligomers formed by Pmp21 exhibit an amyloid-like character (Luczak et al., 2016) that is very similar to the amyloid fibrils formed by Aβ_42_, a protein fragment which plays a vital role in Alzheimer’s disease (Gu and Guo, 2013). Taken together, these converging data, in combination with results obtained with structure-prediction tools, suggest that all Pmp PDs, including rD72, form hMW oligomers that are made up of protofibril structures with amyloid-like properties.

Using rD72 bound to HEp-2 cells as bait in pulldown assays, we identified CLU as a direct interaction partner via mass spectrometry (**Figures 2**, **3**). CLU is a ubiquitously expressed protein present in nearly all human tissues, and occurs in multiple isoforms, including a secreted isoform, sCLU, that is found in the extracellular space (Gross et al., 2023). Secretion of CLU generally occurs from vesicles via the secretory pathway (Nizard et al., 2007). Once outside the cell, sCLU acts in chaperone-like fashion to clear the extracellular space of misfolded proteins that might otherwise give rise to unstructured, amorphous or amyloid aggregates (Humphreys et al., 1999; Poon et al., 2000; Wilson and Easterbrook-Smith, 2000). Thus, sCLU is associated with several proteinopathies, including Alzheimer´s disease (Satapathy and Wilson, 2021). In Alzheimer’s disease, Aβ peptides accumulate in the extracellular space of brain cells and eventually oligomerize and aggregate, forming amyloid fibrils. sCLU is known to bind to Aβ oligomers in the brain, thus preventing their aggregation and inducing the clearance of Aβ via transportation across the blood-brain-barrier (Howlett et al., 2013; Madadi et al., 2019). The endocytic mechanisms triggered by sCLU that enable uptake of cargo and its delivery to lysosomes for degradation have not yet been fully identified. However, it was recently demonstrated that sCLU participates in the chaperone- and receptor-mediated extracellular protein degradation (CRED) pathway for aberrant extracellular proteins, and that sCLU-cargo complexes bind to heparan sulfate (HS) receptors on cells via electrostatic interactions (Itakura et al., 2020).

Our data indicate that sCLU binds directly to soluble rD72, which has been shown to form hMW oligomers that probably have amyloid-like characteristics. Hence, we suggest that the sCLU-rD72 interaction is based on rD72’s capacity to form amyloid-like aggregates, which are natural targets of sCLU. Importantly, alteration of the level of sCLU in the ccm either by replacing spent medium with fresh medium shortly before infection or by immunodepletion of sCLU from 2-day-old ccm, prior to infection with *Ctr* EBs, results in an approximately 30% reduction in the infection rate in the absence of sCLU (**Figure 4**). Based on these experiments, we cannot formally rule out the possibility that other components of the cell culture medium directly or indirectly might also have an influence on the infection rate. Most likely, however, our data suggest that sCLU promotes *Ctr* infectivity.

Furthermore, we would also like to postulate that sCLU not only binds to soluble recombinant PmpD, but also to PmpD that is localized on the surface of EBs. All *Ctr* Pmp proteins act as adhesins and are essential for infection (Becker and Hegemann, 2014). Several Pmp proteins form homo and hetero oligomers with a similar fibrillar structure *in vitro* (Favaroni and Hegemann, 2021; Luczak et al., 2016). For all Pmp passenger domains, AlphaFold predicts a highly similar beta-helical domain structure, which allows for an oligomerization (Cervantes et al., 2024). Based on this, we suspect that sCLU could also bind other, possibly even all Pmps (Favaroni and Hegemann, 2021; Cervantes et al., 2024). This must be tested in further experiments.

Previous work has shown that pre-incubation with soluble rPmpD (and all other rPmps) blocks subsequent infection. However, in these experiments the 2-day-old ccm (enriched in sCLU) was replaced with fresh ccm (lacking sCLU) prior to infection with *Ctr* (Becker and Hegemann, 2014). Moreover, the slight increase in infectivity obtained in approach (iii) in **Figure 4**, confirms published data, which have shown that incubation of infectious EBs with adhesion-competent D72 can functionally replace the naturally exposed adhesive structures, probably because rD72 may be able to bind to the EB cell surface alone as well as in a complex with CLU (Favaroni and Hegemann, 2021). Thus, future experiments will have to clarify how soluble recombinant PmpD mechanistically affects the CLU-dependent *Ctr* infection.

It is at present unclear but likely, that other Pmp proteins can also interact with sCLU. *In vitro*, Pmp proteins have the capacity to interact with themselves and with other Pmps forming functional, polyadhesive, homo- and heteromeric autotransporter complexes (Favaroni and Hegemann, 2021). This makes a PmpD analysis by testing a *pmpD* gene deletion strain challenging. Furthermore, although a PmpD knock-out is available it has been generated in serovar D while our study was performed with the PmpD protein from *Ctr* serovar E (Kari et al., 2014). There is currently no protocol for the transformation of *Ctr* serovars of the urogenital tract. To date, all interventions in the *Ctr* genome have been performed on the L2 serovar strain. The serovar-specific differences make the transfer of data between serovars difficult. We also did not attempt to genetically inactivate the CLU gene because clusterin is a ubiquitously expressed glycoprotein that is involved in a whole range of biological processes including lipid transport, membrane recycling, regulation of apoptosis and inhibiting formation of the complement-mediated membrane attack complex (MAC) (Satapathy and Wilson, 2021). The consequences of CLU inactivation on a chlamydial infection could therefore be direct and/or indirect, which would make their assessment impossible.

Our model is that EBs may be treated as cargo by sCLU, thus facilitating their internalization by the host cell, similar to the mechanism of Aβ clearance from the extracellular space via the CRED pathway. Uptake into host cells may be mediated by heparan sulfate receptors. In addition, the involvement of a co-receptor in facilitating endocytic uptake of the EB by CRED seems likely. Previous studies on various bacterial and viral pathogens that make use of heparan sulfate for host-cell entry have shown that they also rely on co-receptors to promote internalization into the host cell via endocytic processes (Yayon et al., 1991; Liu and Thorp, 2002; Pillay et al., 2016). Indeed, heparan sulfate is already known to be the host receptor for the *Ctr* adhesin OmcB (Mölleken and Hegemann, 2008). Interestingly, blocking adhesion of *Ctr* EBs to HEp-2 cells with heparin (a heparan sulfate analogue) reduces binding by approximately 95%, while addition of rOmcB reduced adhesion of EBs by about 70%. These differences may be explained by the fact that heparin blocks binding of both OmcB and sCLU, while blocking by rOmcB only blocks its specific binding sites on heparan sulfate (Mölleken and Hegemann, 2008).

After sCLU-triggered internalization, the endocytic vesicle is typically designated for lysosomal degradation (Kounnas et al., 1995; Itakura et al., 2020). However, chlamydia avoid this fate, most probably through the tight regulation of Rab GTPase recruitment to the early inclusion membrane, which mediates the transition from the early endosome to a slowly recycling endosome, thus providing a protective “inclusion”, within which the chlamydial development cycle can proceed (Mölleken and Hegemann, 2017). While sCLU recruitment and the CRED pathway may facilitate EB uptake, a possible inhibitory effect of CLU on the terminal complement pathway must also be considered, as CLU has been shown to be associated with EBs of *Ctr* serovar L2 (Partridge et al., 1996; Hallstrom et al., 2015; Fox and Parks, 2022; Lausen et al., 2021).

In conclusion, our data support a unique adhesion and invasion strategy driven by the interaction of *Ctr* PmpD (and very likely all other Pmps) with sCLU. By binding to sCLU, *Ctr* may hijack a host component to facilitate its own uptake into the cell. Thus, sCLU may be crucial for the early *Ctr* infection process.

## Materials and methods

### Antibodies and reagents

The following primary antibodies were used: anti-clusterin α-chain (Santa Cruz, sc-5289), anti-penta-His (Qiagen, #34660), anti-β-actin (Thermo Scientific, MA5-15739) and anti-MOMP (Bio-Rad, #1990-0804). The secondary antibodies – anti-mouse, coupled to alkaline phosphatase, and anti-goat, coupled to Alexa Fluor™ 594 – were purchased from Thermo-Fisher Scientific.

### Bacterial strains and cell lines

The *Saccharomyces cerevisiae* strain CEN.PK2 was used for cloning steps. *E. coli* XL1 blue (Stratagene) and Origami (Novagen) were used for plasmid amplification and protein expression, respectively.

HEp-2 cells (ATCC: CCL-23) were cultured in cell culture medium (ccm), composed of DMEM medium (Thermo Scientific, Waltham, MA, USA) supplemented with 10% heat-inactivated FCS, MEM vitamins and non-essential amino acids (Thermo Scientific, Waltham, MA, USA), and the antibiotics amphotericin B and gentamycin (Life Technologies).

*Ctr* serovar E (DK-20) (London) (NCBI Accession Number: CP015304.1) was propagated in HEp-2 cells in cell culture medium supplemented with 1.2 μl/ml cycloheximide (Sigma), purified by centrifugation and stored in SPG buffer (220 mM sucrose, 3.8 mM KH_2_PO_4_, 10.8 mM Na_2_HPO_4_, 4.9 mM L-glutamine), as previously described (Jantos et al., 1997).

### Cloning, protein expression and purification

Cloning steps were carried out by *in-vivo* homologous recombination in *S. cerevisiae*. The DNA fragment of *pmpD* encoding the D72 protein variant (residues D68-A761) was amplified via PCR from *Ctr* serovar E (DK20) genomic DNA, and cloned into the expression vector pKM32 (generating an N-terminal 10xHis-fusion) (Mölleken et al., 2010). Plasmids were amplified in *E. coli* XL1 blue and the sequence was verified prior to use.

Expression of the His-tagged protein was carried out in the *E. coli* Origami strain. Proteins were purified using Ni-NTA agarose (Thermo Scientific) and dialysed in phosphate-buffered saline (PBS) (10 mM Na_2_HPO_4_, 1.8 mM KH_2_PO_4_, 137 mM NaCl, 2.7 mM KCl, pH 7.4).

### Immunoblotting and Coomassie Blue staining

SDS-PAGE and immunoblotting were performed according to the standard protocol described by Sambrook and Maniatis et al (Sambrook et al., 1989). His-tagged recombinant proteins were detected with monoclonal anti-His antibodies and clusterin was detected with anti-clusterin α-chain antibodies. Both were visualized with AP-conjugated antibodies. SDS gels were stained with Coomassie Brilliant Blue G250 (Serva).

### Blue-Native PAGE

Blue-Native PAGE analysis was performed using the Native PAGE Novex 3-12% Bis-Tris gel system, following the manufacturer’s protocol (Thermo Scientific). Proteins were visualized by staining the gels with Coomassie Brilliant Blue G250 (Serva).

### Size-exclusion chromatography

Recombinantly expressed proteins dissolved in PBS (10 mM Na_2_HPO_4_, 1.8 mM KH_2_PO_4_, 137 mM NaCl, 2.7 mM KCl, pH 7.4) were analysed on a Superose 6 Increase 3.2/300 column (Cytivia) at a flow rate of 0.05 ml/min, and 0.1-ml fractions were collected. All runs were performed at 4°C. The void volume was estimated as described in the manufacturer’s instructions.

### Adhesion assay

Confluent HEp-2 cells were grown at 37°C and specific concentrations of soluble recombinant protein was added to the ccm in a total volume of 250 µl. Binding was allowed to proceed for 1 h at 4°C. After extensive washing, cells were lysed in phospho-lysis buffer (1% NP-40, 1% Triton X-100, 20 mM Tris/HCl, 150 mM NaCl, 1 mM Na_2_VO_4_). The lysate subjected to SDS/PAGE and immunoblotting using anti-His antibodies for the quantification of recombinant proteins. Anti-β-actin antibody served as the internal loading control.

### Pulldown assays

Confluent HEp-2 cells were grown in 25-cm² flasks, and soluble recombinant His-tagged proteins (2 µM) were mixed with the overlying ccm (without FCS and antibiotics) to a total volume of 3 ml. Binding was allowed to proceed for 1 h at 4°C, then cells were washed three times with HBSS (Gibco™) and lysed with phospho-lysis buffer (1% NP-40, 1% Triton X-100, 20 mM Tris/HCl, 150 mM NaCl, 1 mM Na_2_VO_4_). Lysate was centrifuged at 1000 rpm and the soluble fraction was loaded on Ni-NTA agarose. His-tagged proteins and their bound host-cell binding partners were eluted with PBS containing 500 mM imidazole. Proteins enriched in the elution fraction were identified by mass spectrometry.

To verify the direct interaction between sCLU and rD72, an *in vitro* pulldown assay was performed, using rD72 as bait. HEp-2 cells were seeded subconfluently and grown for 2 days to confluence. This ccm carrying secreted CLU was collected (without HEp-2 cells) and incubated with rD72 for 1 h at 4°C followed by the *in vitro* pulldown assay. The protein solution was loaded on Ni-NTA agarose and the His-tagged rD72 and its bound host-cell binding partner were eluted with PBS containing 500 mM imidazole. The protein composition of the individual fractions of the pulldown assay was probed by Western blotting, using anti-His and anti-clusterin α-chain antibodies.

### Quantitative mass-spectrometric analysis of rD72 host-cell binding partners

Eluates from His-PmpD pulldown assays were prepared for mass spectrometric analysis by in-gel digestion with trypsin, essentially as described earlier (Grube et al., 2018).

Briefly, eluates were fractionated in a polyacrylamide gel, proteins were then reduced with dithiothreitol, alkylated with iodoacetamide and digested overnight with 0.1 µg trypsin. Peptides were extracted from the gel and subsequently purified by solid-phase extraction (HLB µ-elution plate, Waters) according to the manufacturer’s instructions. Finally, peptides were dried in a vacuum concentrator, resuspended in 17 µl 0.1% (v/v) trifluoroacetic acid and analyzed by liquid-chromatography-coupled mass spectrometry as described previously, with the modifications mentioned below (Prescher et al., 2021).

First, peptides were trapped on a 2-cm long pre-column, then separated with the aid of a 1-h gradient on a C18 analytical column (25 cm long) using an Ultimate 3000 rapid separation liquid chromatography system (Thermo Fisher Scientific). Peptides were then injected into an QExactive plus (Thermo Fisher Scientific) mass spectrometer online coupled by a nano-source electrospray interface. The mass spectrometer was operated in data-dependent positive mode. Survey spectra were recorded with following settings: resolution 140000, automatic gain control target 3000000, maximum ion time 50 ms, scan range 200-2000 *m*/*z*, profile mode. Up to 20 precursors were selected by the quadrupole of the instrument (4 m/z isolation window), fragmented by higher-energy collisional dissociation (normalized collision energy: 30), and fragment spectra were recorded in the orbitrap analyzer: resolution 17500, automatic gain control target 100000, maximum ion time 50 ms, scan range 200-2000 *m*/*z*, centroid mode. Fragmented peptides were excluded for the next 10 seconds.

Peptide and protein identification was carried out with MaxQuant version 2.1.3.0 (MaxPlack Institute for Biochemistry, Planegg, Germany) with standard parameters, if not stated otherwise. Protein sequences from *Homo sapiens* (79038 entries of UP000005640, UniProt knowledge base, downloaded on 18^th^ January 2022), as well as one entry for PmpD, were used, and the match between runs function and label-free quantification (LFQ) enabled.

Identified proteins were filtered (contaminants, “identified by site”, reverse hits and proteins only identified with one peptide removed) and only proteins identified with at least four valid values in one experimental group were considered for statistical analysis on log_2_ transformed LFQ-intensities. Missing values were imputed with values drawn from a downshifted (1.8 standard deviations (s.d.)) normal distribution (0.3 s.d.) and differentially abundant proteins between the two groups (PBS, PmpD) determined by the significance analysis of microarray method using an FDR of 5% and S_o_ of 0.1 (Tusher et al., 2001).

### Infection assay

For all infection assays, cells were seeded at sub-confluent density in 24-well plates and grown in cell culture medium (ccm) to confluence for two days at 37°C. For approach (i), chlamydial EBs (MOI = 10) were added to the 2-day-old ccm covering the confluent cell layer and incubated for 2 h at 37°C. For approach (ii), the confluent cell layer was washed three times with HBSS, and then fresh ccm, supplemented with EBs (MOI = 10) was added and incubated for 2 h at 37°C. For approach (iii), soluble recombinant rD72 was added to the 2-day-old ccm covering the confluent cell layer. After 1 h of incubation at 37°C, chlamydial EBs (MOI = 10) were added and incubated for 2 h at 37°C. For approach (iv), the 2-day-old ccm used for cell growth was isolated and cells were washed three times with HBSS. From the isolated ccm, sCLU was extracted via immunoprecipitation using Protein-G agarose (Merck) and an anti-clusterin α-chain antibody, following the manufacturer’s protocol. The flow-through fraction depleted in sCLU was then added to HEp-2 cells, and chlamydial EBs (MOI = 10) were added and incubated for 2 h at 37°C.

For all four approaches, after the initial 2 h of incubation with EBs without centrifugation, the ccm containing non-bound chlamydial EBs was removed and fresh ccm supplemented with cycloheximide (1.2 µl/ml) was added to the cells. Infection was allowed to proceed for 24 h at 37°C, before the cells were prepared for immunofluorescence microscopy. The presence of sCLU in the ccm used for the different approaches was monitored by immunoblotting using an anti-clusterin α-chain antibody.

### Immunofluorescence microscopy

Infected cells were fixed with 3% para-formaldehyde for 10 min at RT, washed three times with PBS, and permeabilized with 96% methanol (-20°C) at room temperature. The primary antibody (anti-MOMP, 1:500) was diluted in bufferX (5% BSA, 0.5% Triton-X 100, 0.2% Tween20) and incubated for 1 h at RT. Cells were washed three times with PBS, and the secondary antibody (anti-goat coupled to Alexa594, 1:1000), diluted in buffer X, was incubated for 60 min at RT. Cells were then washed three times and DAPI was used to visualize DNA. Microscopy was performed using a Nikon Eclipse Ti-E C2 confocal microscope (DS-Qi1MC Camera) and confocal images were generated using NIS Element software (Nikon) and quantified using ImageJ.

### Statistical analysis

Graphics were created in OriginPro v.2021b (OriginLab). The data represent the means ± s.d. A 1-way ANOVA (with multiple comparisons) was used to compare all four groups shown in **Figure 4D**. Images were prepared using the open-source software Inkscape (www.inkscape.org).

## Data Availability

All relevant data is contained within the article. The original contributions presented in the study are included in the article/**Supplementary Material**; further inquiries can be directed to the corresponding author.
