# Supplementary material for: The secreted host-cell protein clusterin interacts with PmpD and promotes Chlamydia trachomatis infection
Source: Front Cell Infect Microbiol. 2025 Jan 27;14:1519883. doi: 10.3389/fcimb.2024.1519883 (PMC11807975; doi:10.3389/fcimb.2024.1519883)
Supplement: Supplementary file 1 [file DataSheet1.pdf]

Supplementary Information

The secreted host-cell protein clusterin interacts with PmpD and promotes *Chlamydia trachomatis* infection

Fabienne Kocher, Johannes H. Hegemann

Supplementary Contents

Supplementary Figures.. 2
Supplementary Table 7
References 8

Supplementary Figures

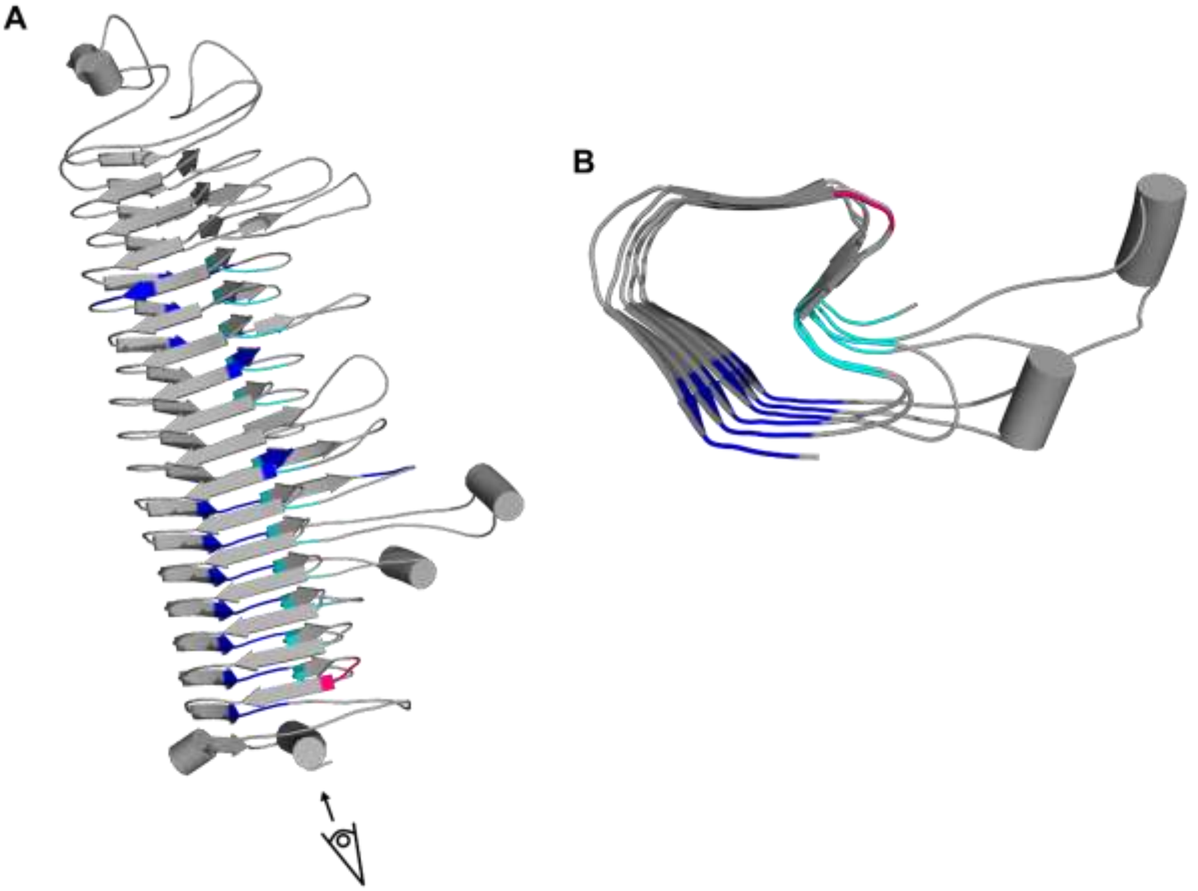

Supplementary Figure 1: Predicted 3D-model for the D72 fragment of *Ctr* PmpD

(A) The predicted structure of rD72, a fragment of *Ctr* PmpD spanning residues 68-761, indicates a β-helical structure. The FxxN and GGA(I,L,V) motifs are highlighted in dark and light blue, respectively. The structure was generated by RoseTTAFold [1]. **(B)** Longitudinal view of the structure depicted in **(A)**, showing the FxxN and GGA(I,L,V) motifs stacked on top of one another.

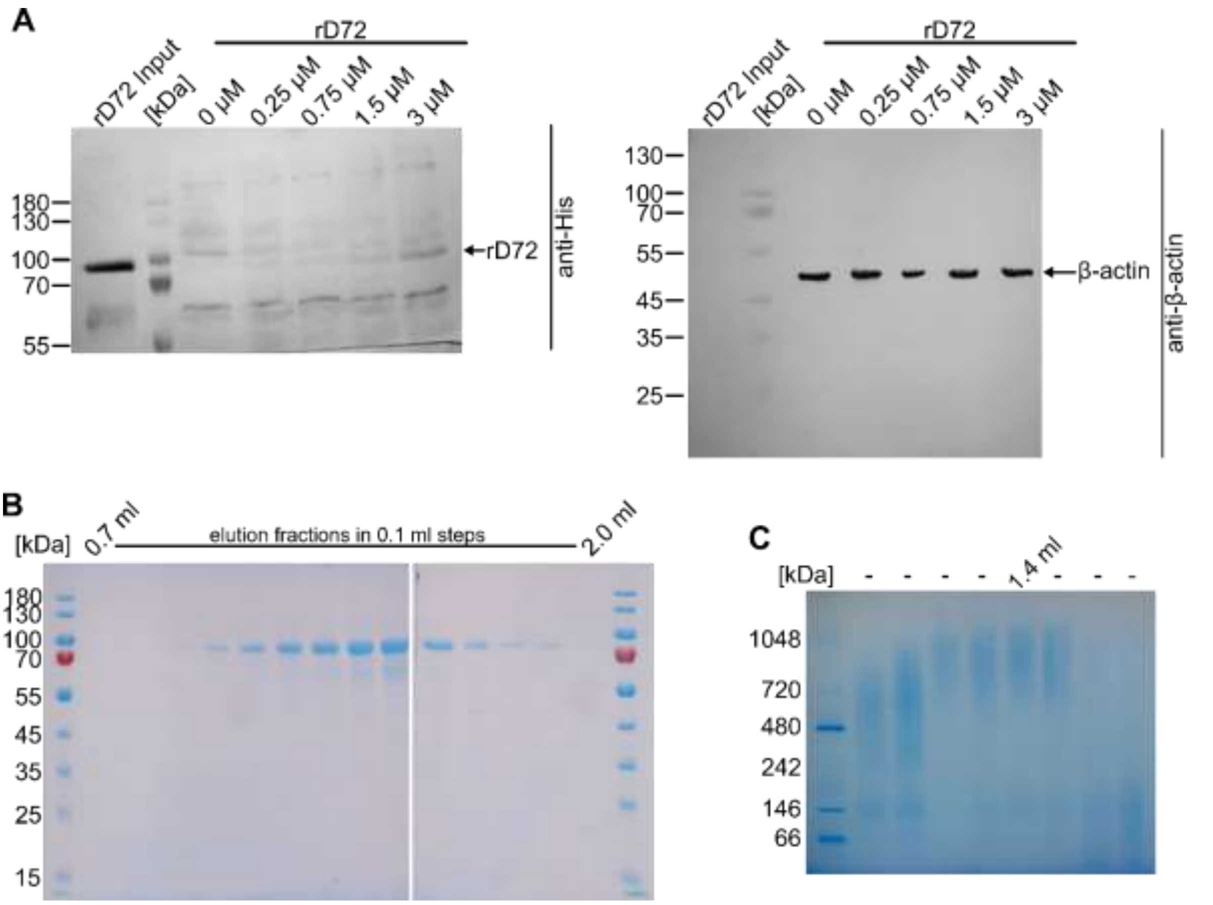

Supplementary Figure 2: Uncropped gels and blots from Figure 1. (A) Immunoblots from Fig. 1B depicting the concentration-dependent affinity of rD72 for HEp-2 cells. rD72 was detected using an antibody against the penta-His tag. Lane 2 shows a molecular-weight ladder. The blot on the right shows an internal loading control, as visualized with anti-β-actin antibodies. **(B)** Images of gels for Fig. 1C, showing the individual SEC elution fractions. On the left is a Coomassie stained SDS-PA gel, which depicts elution fractions obtained by SEC analysis. The panel on the right shows fractions obtained by Blue Native-PAGE.

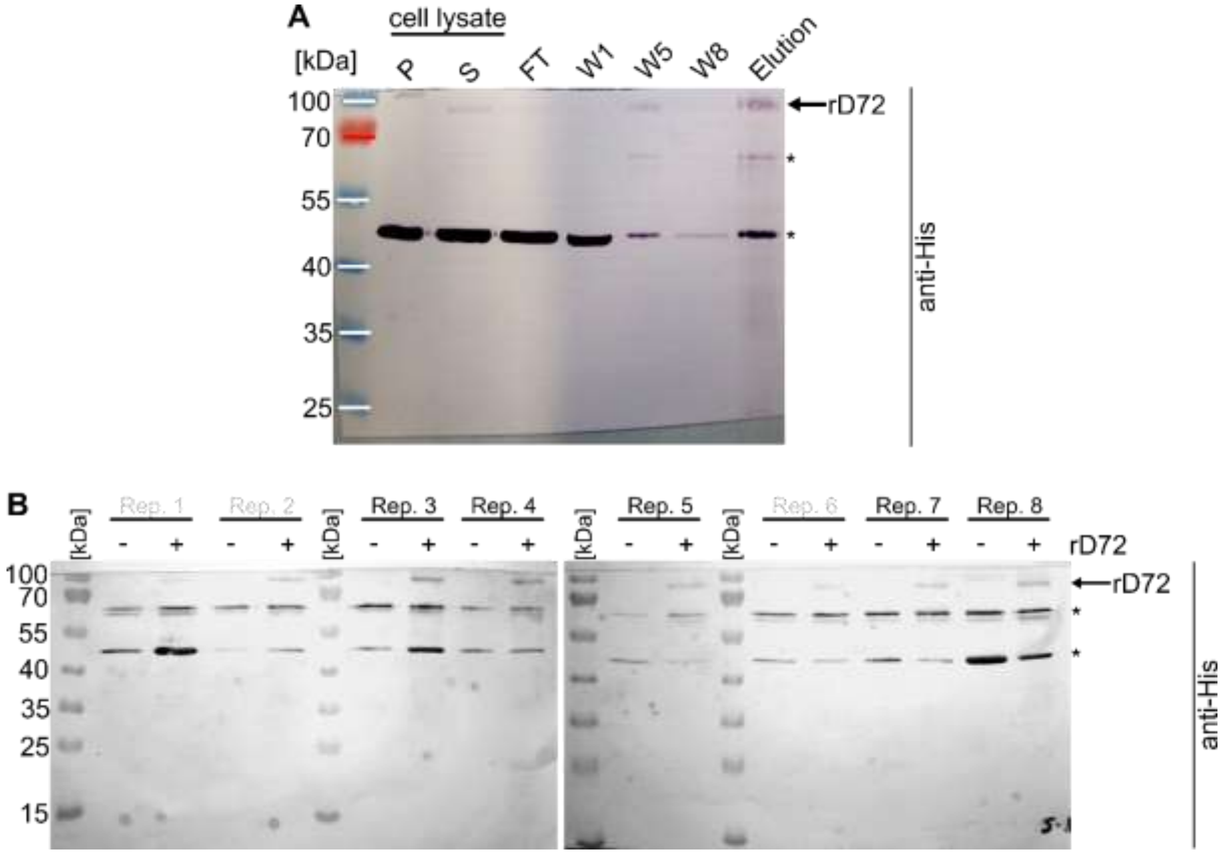

Supplementary Figure 3: Uncropped blot for Fig. 2A and biological replicates generated for mass spectrometry. **(A)** Immunoblot picture from Fig. 2A used to monitor the pulldown assay with rD72 as bait on epithelial cells, visualized with anti-His antibodies. Nonspecific bands are indicated by asterisks. **(B)** The pulldown elution fractions of the negative controls (-) and the test samples (+) were analysed by Western blotting and probed with anti-His antibodies. Replicates used for mass spectrometry analysis are marked in black; replicates which were not processed further are indicated in grey.

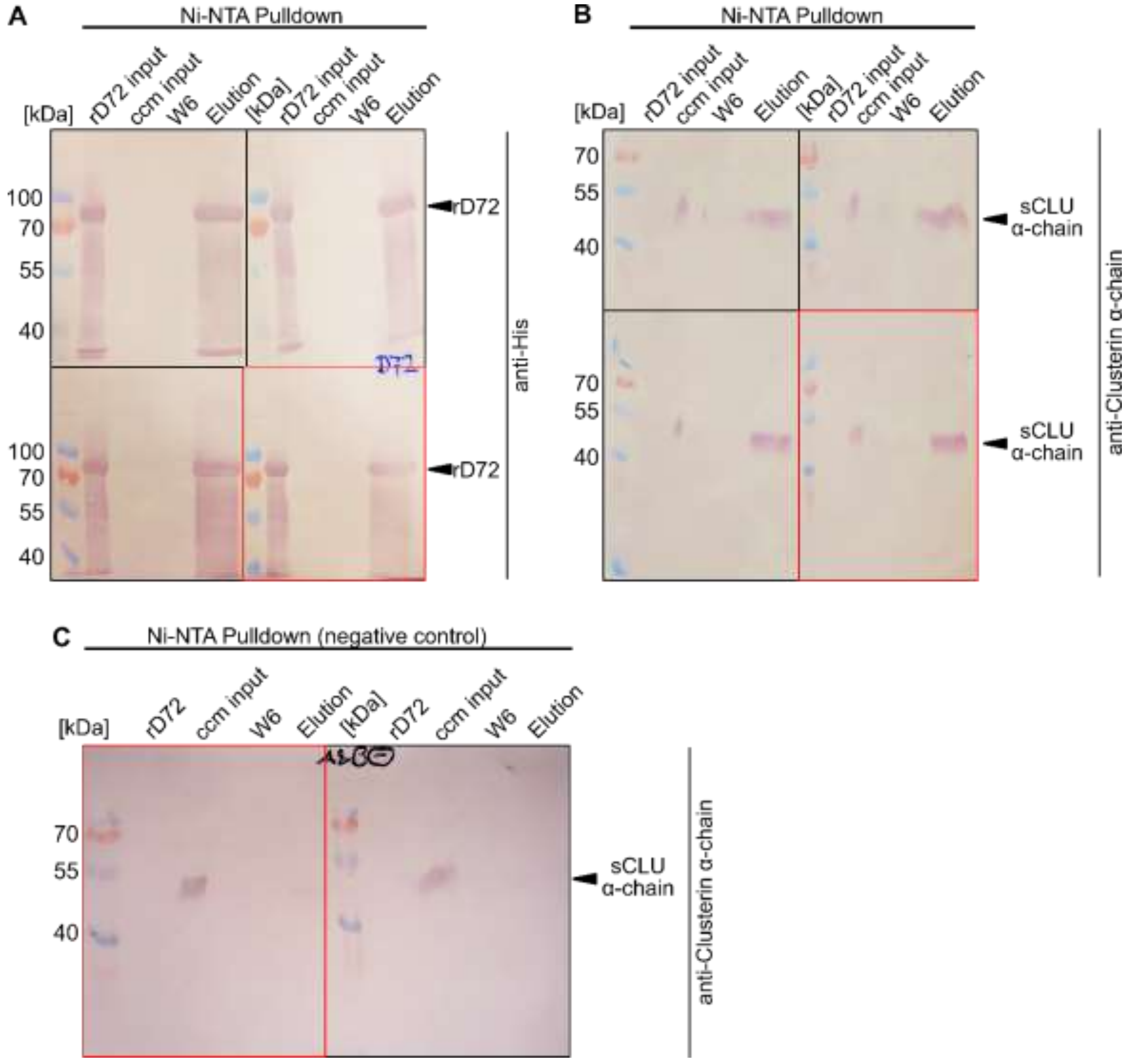

Supplementary Figure 4: Replicates and uncropped blots of the pulldown assay shown in Fig. 3. Panels **(A)** and **(B)** depict Ni-NTA pulldown assays in which rD72 was used as bait and incubated with cell culture medium isolated from epithelial cells grown for two days. The individual pulldown assay fractions were analysed by Western blot, using **(A)** an anti-His antibody or **(B)** an anti-Clusterin α-chain antibody (W6 = wash 6). The boxes in **(A)**, **(B)** and **(C)** mark the four replicates. The red boxes indicate the blot shown in Fig. 3B. **(C)** Replicates of the negative control, to which no rD72 was added (W6 = wash 6). The boxes indicate the two replicates, the red box shows the blot shown in Fig. 3C.

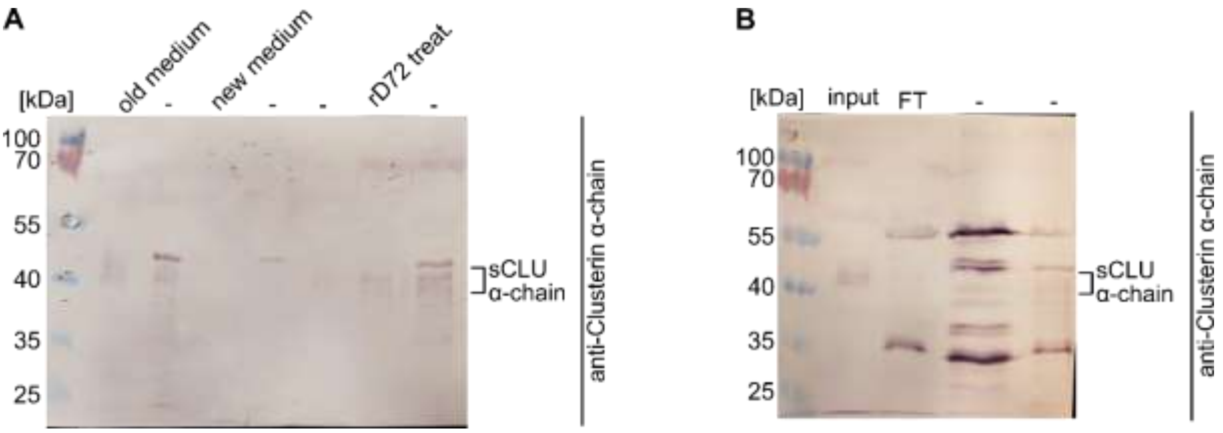

Supplementary Figure 5: Uncropped blot for Figure 4. (A) Western blot from Fig. 4B depicting levels of the sClu alpha-chain detected by anti-clusterin-α antibodies in the different experimental approaches (i-iii) described in Fig. 4A. **(B)** Western blot from Fig. 4B, which shows the relative intensity of the sClu alpha-chain detected in the fourth experimental approach, described in Fig. 4A, using anti-Clusterin-α antibodies. (FT = flow through).

Supplementary Table

Supplementary Table 1. Vectors and oligonucleotides for cloning *pmpD* fragments.

Pmp fragment	Vector	Primer sequence (5' to 3')
PmpD (D72)	pKM32 [2]	GAAATTAACTATGAGAGGATCTCACCATCACCATCACCATCACCATCACCATGATAGTCAGGCTGAAGGACAG
		GGAGTCCAAGCTCAGCTAATTAAGCTTGGCTGCAGGTCGACTAAGCTTGATTAGCTGCAGTAATAAAAC

Supplementary Table 2. List of significantly enriched host cell proteins detected by mass spectrometry analysis.

Protein ID	Protein name	Gene name	MW [kDa]	Localization
P04792	Heat shock protein beta-1	HSPB1	23.704	cytosolic
Q9H936; Q9H1K4	Mitochondrial glutamate carrier 1 & 2	SLC25A22; SLC25A18	34.47	mitochondrial
Q9NZJ7	Mitochondrial carrier homolog 1	MTCH1	43.121	mitochondrial
P10909	Clusterin	CLU	52.494	intra- & extracellular
P05141	ADP/ATP translocase 2	SLC25A5	32.852	mitochondrial
Q9UJS0	Calcium-binding mitochondrial carrier protein Aralar2	SLC25A13	74.175	mitochondria

References

1. Baek M, DiMaio F, Anishchenko I, Dauparas J, Ovchinnikov S, Lee GR, et al. Accurate prediction of protein structures and interactions using a three-track neural network. Science. 2021;373(6557):871-6; doi: 10.1126/science.abj8754
2. Mölleken K, Schmidt E, Hegemann JH. Members of the Pmp protein family of Chlamydia pneumoniae mediate adhesion to human cells via short repetitive peptide motifs. Mol Microbiol. 2010;78(4):1004-17; doi: 10.1111/j.1365-2958.2010.07386.x
